# Farmer to pharmacist: curcumin as an anti-invasive and antimetastatic agent for the treatment of cancer^1^

**DOI:** 10.3389/fchem.2014.00113

**Published:** 2014-12-23

**Authors:** Debasish Bandyopadhyay

**Affiliations:** Department of Chemistry, The University of Texas-Pan AmericanEdinburg, TX, USA

**Keywords:** cancer, carcinoma, adenocarcinoma, invasion, metastasis, angiogenesis, curcumin, polyphenol

## Abstract

A huge number of compounds are widely distributed in nature and many of these possess medicinal/biological/pharmacological activity. Curcumin, a polyphenol derived from the rhizomes (underground stems) of *Curcuma longa* Linn (a member of the ginger family, commonly known as turmeric) is a culinary spice and therapeutic used in India for thousands of years to induce color and flavor in food as well as to treat a wide array of diseases. The origin of turmeric as spice and folklore medicine is so old that it is lost in legend. Curcumin has many beneficial pharmacological effects which includes, but are not limited with, antimicrobial, anti-inflammatory, antioxidant, antiviral, antiangiogenic, neurodegenerative diseases such as Alzheimer disease, and antidiabetic activities. Most importantly curcumin possesses immense antitumorigenic effect. It prevents tumor invasion and metastasis in a number of animal models, including models of lung, liver, stomach, colon, breast, esophageal cancer etc. Invasion and metastasis are considered as one of the hallmarks in cancer biology. The pertinent recent applications of curcumin as anti-invasive and antimetastatic agent in *in vitro* and *in vivo* and *ex vivo* studies as well as associated molecular mechanisms have been discussed in this review. Curcumin has also demonstrated the ability to improve patient outcomes in clinical trials.

*Cancer* is neither a single nor a modern disease. In human, more than 200 different types of cancer are possible depending upon the types of tissue and/or cell. The description of cancer was found in many ancient literatures including Egyptian “Edwin Smith” and “George Ebers” papyri, written between the 3000 BC^2^ and 1500 BC (Faguet, 2014). The “Edwin Smith” papyrus refers to 8 cases of ulcers (tumors) of the breast that were removed by cauterization with a tool called “the fire drill.” It describes the disease as: “there is no treatment” (Faguet, 2014). A scientific look into the fossils of over 700 dinosaurs confirmed the existence of bone cancer. Osteosarcoma (growths suggestive of the bone cancer) was also been noticed in the ancient human mummies in Egypt. The word *oncos* (in Greek, it means *swelling*) was introduced by a Greek physician Galen (130–200 AD) to define tumors. Successively the term *oncology* was developed. The ancient Greek physician Hippocrates (460–370 BC), also known as the “Father of Medicine,” first differentiated the benign and malignant tumors by introducing the terms *carcinos* and *carcinoma* to describe non-ulcer forming (benign) and ulcer-forming (malignant) tumors. The word *carcinoma* in Greek stands for *crab*. Most probably, the disease was symbolized with *crab* because of its finger-like tendency to spread throughout the body. Celsus, an ancient Roman physician (28 BC-50 AD) translated the Greek word *carcinoma* into *cancer* (means *crab* in Latin). Since then, several hundred of years no notable progress on understanding and treatment of cancer was made until 1775 when Percivall Pott (a British physician) noticed that a number of young boys employed as chimney sweeps developed cancer of the scrotum in later life. He suggested the presence of *something* in the soot which caused cancer. Therefore, the concept of carcinogen (cancer causing agent) was developed (Rothschild et al., 1998, 1999, 2003; Faguet, 2014). With the progress of research and consequently with better understanding the meaning of carcinoma has been modified and presently it denotes the cancer arising in epithelial cells that cover external and internal body surfaces; approximately 90% of all human cancers are of this type. Also many other commonly used terms have been developed e.g., sarcoma (cancer arising in mesenchyme-derived tissue supporting tissue) which includes cancers of bone, cartilage, fat, connective tissues and muscle; lymphoma cancer arising in the lymph nodes and tissues of the immune system, leukemia (cancer of the immature blood cells that grow in the bone marrow and tend to accumulate in large numbers in the bloodstream), adenoma (cancer of glandular epithelium, in Latin *adeno* means *gland*) etc.

*Cancer* is the leading cause of death in developed countries and the second leading cause of death (after cardiovascular and related diseases) in developing countries^3^ and accounted for 8.2 million deaths in 2012 among which 70% occur in low- and middle-income countries. According to the World Health Organization the annual cancer cases will rise from 14 million in 2012–2022 within the next two decades^4^^,^^5^. Cancer can spread in the body following two mechanisms: invasion and metastasis. According to National Cancer Institute, invasion denotes “the direct migration and penetration by cancer cells into neighboring tissues” whereas metastasis stands for “the ability of cancer cells to penetrate into lymphatic and blood vessels, circulate through the bloodstream, and then invade normal tissues elsewhere in the body”^6^. To have a clear understanding, these two mechanisms can be compared with the ways fire can spread in a locality. When fire spread from one house to its adjacent house the process can be compared with invasion where cancer tissue/cells invade from one tumor to its adjacent body organ and new tumor is formed in that organ. The spark of fire can also spread from one burning house to a remote house by air (carrier). This can be compared with the migration of cancer tissue/cells carrying out by blood or lymph to another part of the body (for example from lung to liver) and new tumor is formed in that organ. Invasion and metastasis are considered as one of the hallmarks in cancer biology. Although cancer is treated by surgery, radiotherapy, immunotherapy or gene therapy, separately or in combination, still chemotherapy plays crucial role for the treatment of cancer; particularly to inhibit invasion and metastasis. Therefore, there is a continuous need to search new anticancer agents to prevent invasion and metastasis and subsequently to reduce cancer-related mortality. Current research in this field directs to identify potent anti-invasive and antimetastatic novel chemotherapeutics. A qualitative estimate states that about 25% drugs are still directly derived from the Mother Nature and 74-80% of all cancer drugs have their origins in natural products i.e., these drugs are made by natural product modification. Of the 1355 new entities introduced as therapeutics between 1981 and 2010, 71% were natural products or natural product derived compounds (Hasima and Aggarwal, 2012; Newman and Cragg, 2012).

Curcumin (diferuloylmethane), the major constituent of curcuminoids, is present in turmeric which is the dried powered rhizomes (underground stems) of *Curcuma longa* Linn of the Zingiberaceae family. The medicinal use of turmeric (haridra) was indicated in Sushruta Samhita, one of the three fundamental texts of Ayurveda (Indian traditional medicine) in the 6th century BC. Its medicinal uses were also found in Charaka Samhita (300–500 BC). As Indian traditional medicine (Ayurveda) turmeric has been using to treat a broad range of common disorders for over 6000 years (Padhye et al., 2010). In India, turmeric is used as a cooking spice to induce nice yellow-orange color and flavor in curries, pickles and chutneys. It is used worldwide as a color inducing agent as well as preservative in American mustard, mayonnaise, butter and margarine and has been designated as international food additive E100 (Epstein et al., 2010). Turmeric is in the GRAS (Generally Recognized As Safe) list of the US Food and Drug Administration having GRN number 460^7^. This royal spice was introduced to the western world in the 13th century by Marco Polo, one of the early European explorers to the Indian subcontinent (Aggarwal et al., 2007; Basnet and Skalko-Basnet, 2011). Since then India is the highest turmeric producing country in the world (Ploto, 2003; Basnet and Skalko-Basnet, 2011,^8^). Turmeric has at least 76 synonyms listed in the 1999 World Health Organization (WHO) monograph (World Health Organization, 1999). A few popular names are Haridra (Sanskrit), Halood (Bengali), Haldi (Hindi), Kurkum uqdah safra (Arabic), Ukon (Japanese), Chiang Huang (Chinese), Ulgeum (Korean), Kurkuma (German), Safran des Indes (French), kurkumy (Russian), Indian saffron etc. Besides its culinary appeal turmeric has a glorious history of uses as a therapeutic and preventive agent against a wide array of disorders and diseases, either by itself or in combination with other agents. As an ancient household remedy a hot poultice of turmeric powder and slaked lime (*chun-halood* in Bengali) is applied locally to relieve muscular pain and inflammation caused by sprain and injury. In some parts of India, a drink made from fresh turmeric, ginger roots and honey in a glass of hot milk are given to women twice daily after childbirth. A poultice of fresh turmeric paste is also applied to the perineum as wound healing for lacerations in the birth canal (Hatcher et al., 2008). Its traditional uses as strong therapeutic or preventive agents against several human diseases include, but are not limited to, diabetes, fibrosis, asthma, rheumatism, allergies, inflammation, intestinal worms, atherosclerosis, diarrhea, dyspepsia, intermittent fevers, biliousness, cough, sinusitis, constipation, jaundice, urinary discharges, flatulence, leukoderma, amenorrhea, acne, colic inflammation, respiratory ailments, lupus nephritis, irritable bowel syndrome, menstrual difficulties, anorexia, coryza, hematuria, hemorrhage, neurodegenerative such as Alzheimer's disease as well as cancer (Ammon and Wahl, 1991; Ploto, 2003; Chattopadhyay et al., 2004; Salvioli et al., 2007; Hatcher et al., 2008; Ringman et al., 2012; Gupta et al., 2013b). The presence of turmeric is essential in most of the religious ceremonies in Hinduism. Besides the ancient literature, the first scientific report related to the medicinal uses of *Curcuma* was published in 1748 (Loeber and Buechner, 1748; National Cancer Institute, 1996). After 67 years a review detailing the biological and medicinal properties of turmeric (curcumin) was published (Vogel and Pelletier, 1815). In 1937, Oppenheimer reported the medicinal activity of turmeric against biliary diseases (Oppenheimer, 1937). In 1949, Schraufstatter and Bernt reported (Schraufstatter and Bernt, 1949) the antibacterial activity of curcumin and its pharmacological activity to cure eye disease was reported (Chaudhri, 1950) in the following year. Jiang and co-workers evaluated the anticancer activity of four natural products *viz*. camptothecin, harringtonin, cantharidin, and curcumin on human tumor biopsies in an *in vitro* soft agar clonogenic assay system and reported their findings in 1983 (Jiang et al., 1983). They reported curcumin as “relatively ineffective” antitumor agent than camptothecin and harringtonin. Probably this was the first evaluation of curcumin against cancer. Kuttan and colleagues published the anticancer activity of curcumin in 1985 (Kuttan et al., 1985).

The acceptance of traditional medicine is considered as an alternative form of modern health care system. Over the past quarter century there has been growing interest in a possible role of curcumin on various diseases. The research topic “curcumin” in any chemistry/health related search engine hits a huge number of results, including research articles, reviews, communications, patents, books, editorials etc. Comparative results obtained up to December 31, 2013 from the two major chemistry and health related databases *viz*. SciFinder Scholar and PubMed Central are presented in Figure 1.

**Figure 1:**
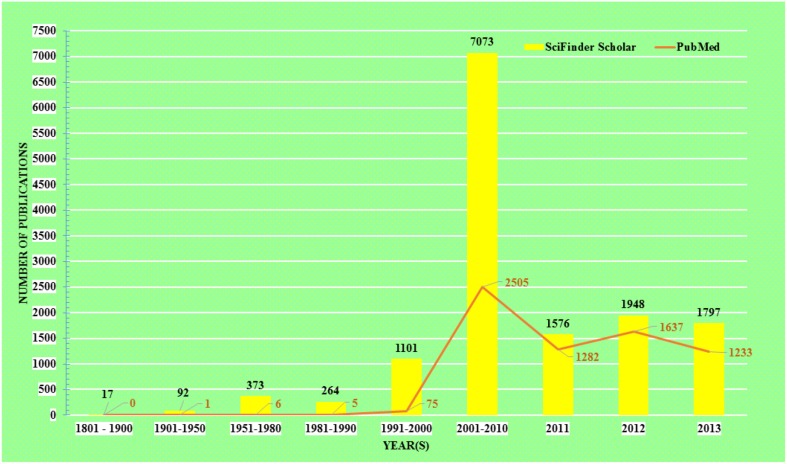
**Comparative literature on research with Curcumin**.

The presence of about 235 compounds have been identified in turmeric of which 109 sesquiterpenes, 68 monoterpenes, 22 diarylheptanoids and diarylpentanoids, 8 phenylpropene, and other phenolic compounds, 5 diterpenes, 4 sterols, 3 triterpenoids, 2 alkaloids, and 14 other compounds. Among the diarylheptanoids 3 are curcuminoids, the major pharmacologically active ingredients of turmeric. Curcumin, the major curcuminoid which constitutes 3–5% of turmeric has been consumed for medicinal purposes for thousands of years (Goel et al., 2008; Gupta et al., 2013b). Two other curcuminoids are demethoxycurcumin and *bis*-demethoxycurcumin (Figure 2). Commercial curcumin is a mixture of three curcuminoids: curcumin (71.5%), demethoxycurcumin (19.4%) and *bis*-demethoxycurcumin (9.1%) (Gupta et al., 2013b).

**Figure 2:**
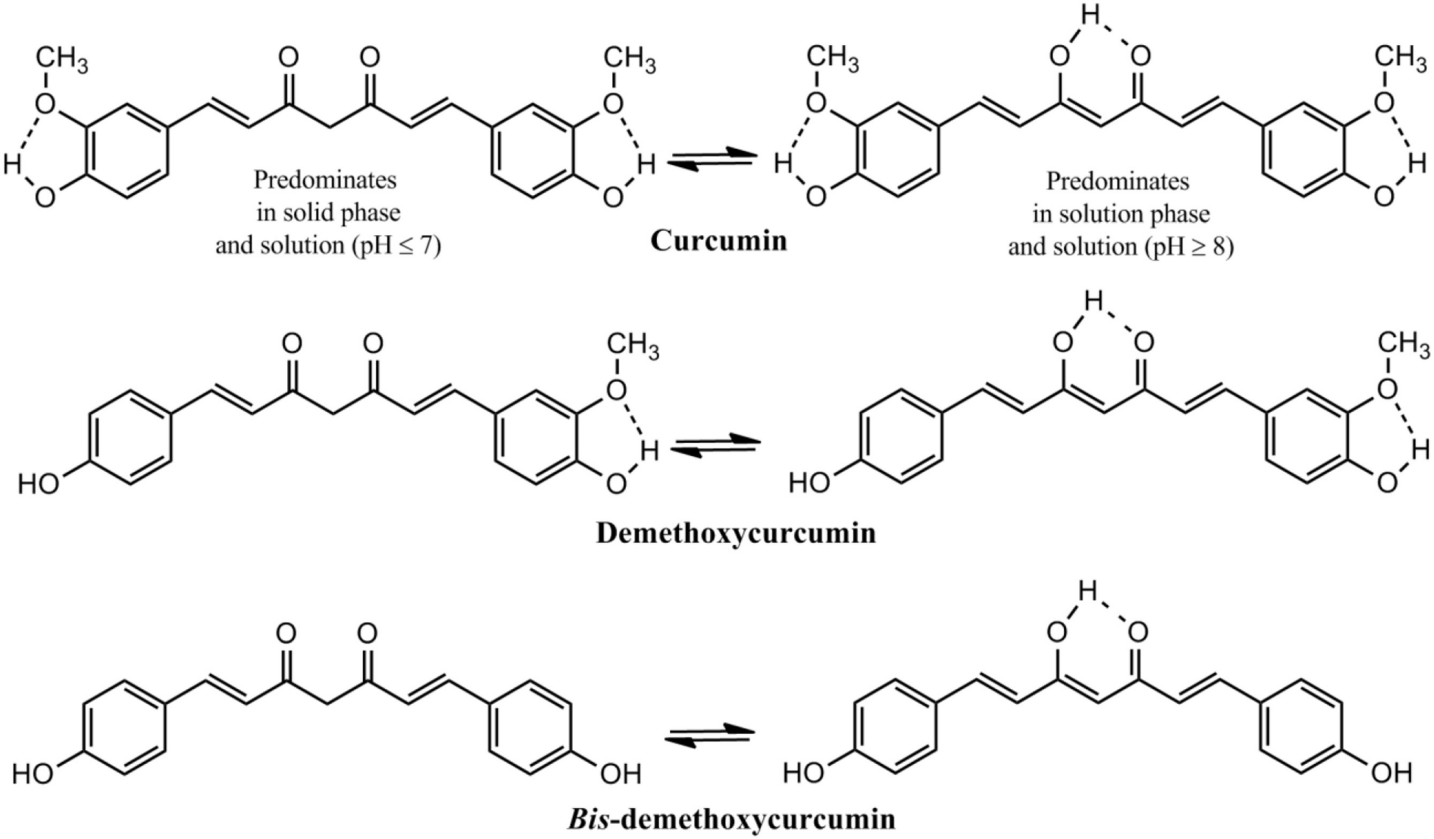
**Structure of Curcuminoids: The active principle in turmeric**.

The isolation of curcumin, the principal constituent of turmeric responsible for its vibrant yellow-orange color, first reported in 1815 (Vogel and Pelletier, 1815) from the plant *Curcuma longa* as “yellow coloring-matter” and named as curcumin. Later, it was found to be a mixture of resin and turmeric oil (Gupta et al., 2012). Curcumin was isolated in 1842 but the chemical formula was not reported (Vogel, 1842; Gupta et al., 2012). The chemical structure of curcumin as diferuloylmethane was identified by Milobedzka et al. (1910) and the first synthesis of curcumin was reported from the same laboratory in 1913 (Milobedzka et al., 1910; Lampe and Milobedzka, 1913; Gupta et al., 2012). In 1953, Srinivasan reported the chromatographic resolution and quantification of curcumin from curcuminoids (Srinivasan, 1953). The chemical structure of curcumin was confirmed in 1973 (Roughley and Whiting, 1973) and a few years ago in 2007, its solution structure (Payton et al., 2007) was reported (Figure 3).

**Figure 3:**
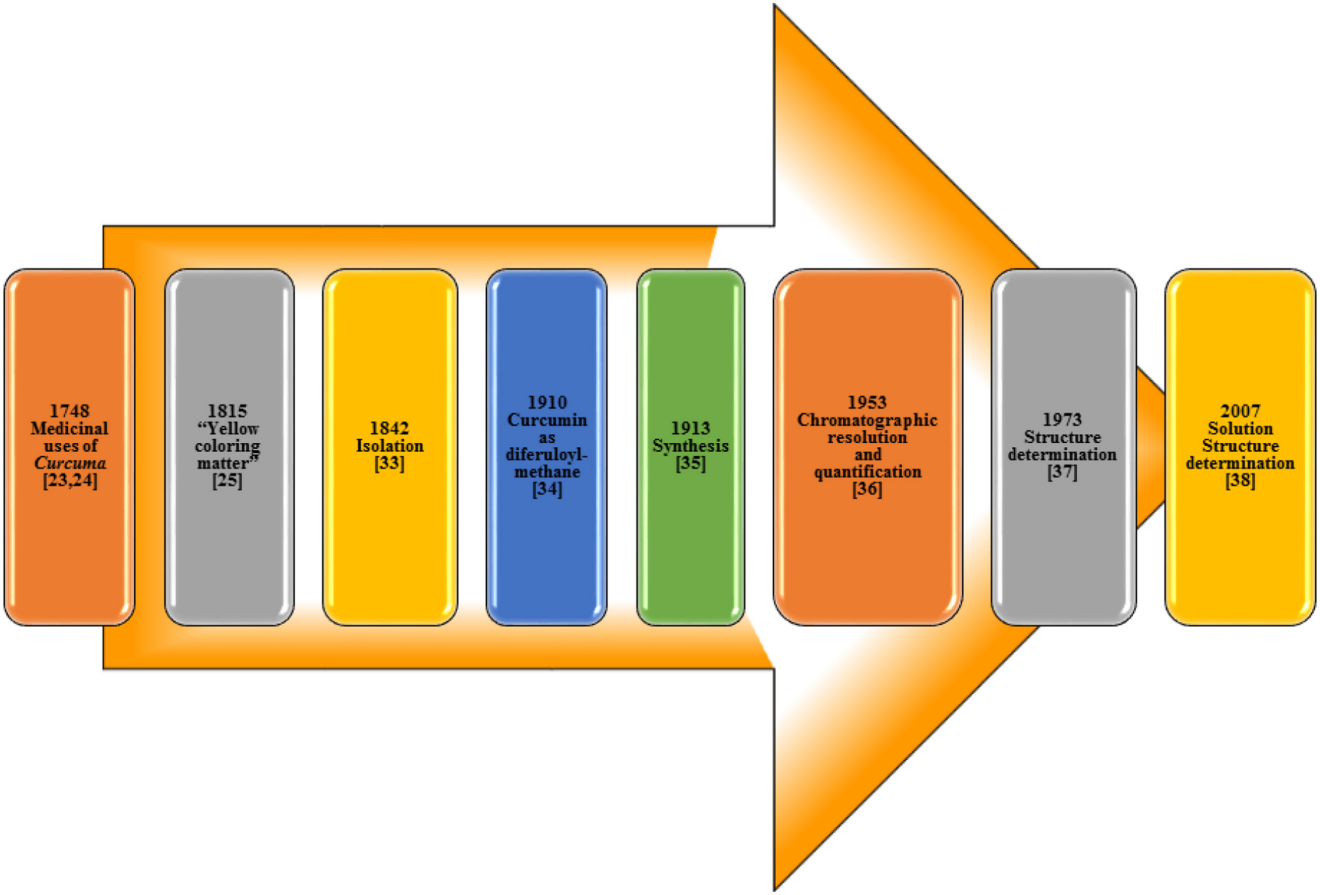
**Milestones in Curcumin research**.

Since the publication of the preliminary investigations on the anticancer activity of curcumin as a promising anticancer agent in the mid-eighties (Jiang et al., 1983; Kuttan et al., 1985), scientists from all over the world have been paying their attention to this novel nutraceutical. It is now well known that cancer is neither a single disease nor a comparatively new disease. It has already been mentioned that invasion and metastasis are two major problems for the treatment of cancer. Curcumin has successfully demonstrated its potential to act as a potent anti-invasive as well as anti-metastatic agent *in vitro*, *ex vivo* and *in vivo* in numerous occasions. It can inhibit cancer cell migration and invasion by manipulating several signaling pathways as described in the sequel.

## Lung cancer

The overall incidence (13.0%) and cancer-related mortality (19.4%) in lung cancer were highest worldwide among all cancers in 2012^4,5^. About 85-90% lung cancers are non-small cell lung carcinoma (NSCLC) and 5-year prevalence is only 5.8% (Vogelstein and Kinzler, 2004). The protein Rac1 (one of the most important small Rho GTPases family proteins) is extensively associated in cytoskeleton rearrangements and cancer cell migration, invasion and metastasis. Curcumin at 10 μ M demonstrated significant inhibitory effect on epidermal growth factor or transforming growth factor β1-induced 801D lung cancer cells migration and invasion. The inhibition of the metastasis ability (at least partly) detected in curcumin-treated cells was due to the inhibition of Rac1/PAK1 signaling pathway and the decreased MMP-2 and MMP-9 expression (Chen et al., 2014). Not only in NSCLC but also in the small cell lung carcinoma (SCLC) cell lines, NCI-H446 and NCI-1688, curcumin at 15 μ M plays notable role to prevent migration, invasion as well as angiogenesis via Janus Kinase-STAT3 signaling pathway. It can inhibit STAT3 phosphorylation to suppress of an array of STAT3 downstream targets that are responsible for colony formation, cell migration and invasion. It also suppresses the expression of proliferative proteins (Survivin, Bcl-XL, and Cyclin B1), and invasive proteins (VEGF, MMP-2, MMP-7, and ICAM-1). Knockdown of STAT3 expression by siRNA was able to induce anti-invasive effects *in vitro*. Curcumin causes G2/M Phase cell cycle arrest in NCI-H446 cells in a time-dependent manner (Yang et al., 2012).

Plectin, a high molecular weight (≈500 KDa) linker protein that organizes the cytoskeleton, can play an important role in the migration and invasion of human NSCLC A549 cells. The down regulation of plectin gene *PLEC1* by siRNA promotes the migration and invasion of the A549 lung cancer cells. The plectin siRNA has been reported (Ha et al., 2011) to mitigate by curcumin. Besides the prevention of plectin siRNA, curcumin demonstrated its ability to inhibit the matrix metalloproteinase (MMP)-2 and 9 as well as VEGF in human lung cancer A549 cells *in vitro*. It is well known that matrix metalloproteinases (MMPs) play a crucial role in the invasion, metastasis and angiogenesis of cancer cells. The down regulation of MMP-2 and -9 occurs through MEKK and ERK signaling pathways in A549 lung cancer cells (Lin et al., 2009). On the other hand, curcumin inhibits the invasion and metastasis of human lung adenocarcinoma (CL1-5) cells through activation of the tumor suppressor DnaJ-like heat shock protein 40 (HLJ1) via activation of the JNK/JunD pathway and by modulating E-cadherin expression (Chen et al., 2008).

## Liver cancer

Liver cancer has been rated as the second leading cause of cancer related deaths (9.1%) worldwide in 2012 with 5-year prevalence of 1.9% ^4,5^. In general, hepatocellular carcinoma is resistant to standard chemotherapy. Surgery or liver transplantation at the early stage may provide longer survival to the patients. However, about 80% of liver cancer patients with advanced stage are not amenable to liver transplantation or surgery and this is the main reason of poor prognosis in liver cancer (Thomas and Abbruzzese, 2005). Vasculogenic mimicry (VM) is considered as one of the major factors in cancer invasion and it denotes the process in which the cancer cells mimic endothelial cells by forming blood channels. The potential of Curcumin on vasculogenic mimicry of human liver adenocarcinoma cells (SK-Hep-1) has been studied *in vitro*. Curcumin inhibited vasculogenic mimicry, decreased cell migration and MMP-9 (matrix metalloproteinase-9) production of the cancer cells. Curcumin exhibited the anti-VM efficacy by down-regulating the Akt (or PKB) and STAT3 signaling pathways (Chiablaem et al., 2014).

It has been stated earlier that the invasion of cancer cells is considered as an important and distinctive step toward metastasis. The blockage of this physiological incident by medicines or supplements/nutraceuticals prolongs the life span of an affected host. A possible involvement of the antioxidant property of Curcumin with its anti-invasive ability was investigated (Kozuki et al., 2001). The efficacy of Curcumin on the proliferation and invasion of the rat ascites hepatoma AH109A cells was evaluated *in vitro* and *ex vivo*. To satisfy this goal, a co-culture system of the hepatoma cells with mesothelial cells derived from rat mesentery was subjected for invasion study. Curcumin reduced the hepatoma slipping motility in a concentration-dependent manner up to 5 μM and afterward maintained the effect up to 20 μM with a minimum impact on cell proliferation. Interestingly, the sera isolated from rats, those consumed Curcumin orally, also inhibited the AH109A cellular invasion when added to the culture medium. Curcumin and curcumin-containing rat sera inhibited the ROS-potentiated invasive capacity by concurrently treating AH109A cells with hypoxanthine, xanthine oxidase and either of Curcumin samples.

High invasive and lymphatic metastasis potential, both *in vitro* and *in vivo*, have been observed in mouse hepatoma cellular carcinoma Hca-F cells. Curcumin successfully down related Caveolin-1 (Cav-1, an important structural component in tumor metastasis which can potentiate the invasive ability by up-regulating CD-147 glycosylation level), and inhibited the phosphorylation of EGFR and the corresponding downstream targets such as MMP-2 and -9 as well as the phosphoinositilde 3-kinase (PI3K)/protein kinase B (Akt), p38 mitogen-activated protein kinase (MAPK), and p44/42MAPK (Wang et al., 2011). Consequently, high level of anti-invasion was noted in Hca-F cells. The upregulation of MMP-9 is much higher in human hepatocellular carcinoma (HCC) SK-Hep-1 cells than HCC Huh-7 cells. Accordingly, SK-Hep-1 tumor is more invasive than the tumor develops from Huh-7 cells. Curcumin at 10 μ M, inhibited the cellular migration and invasion, 17.4 and 70.6% respectively, in SK-Hep-1 cell lines by inhibition of MMP-9 secretion (Lin et al., 1998).

**Figure 4:**
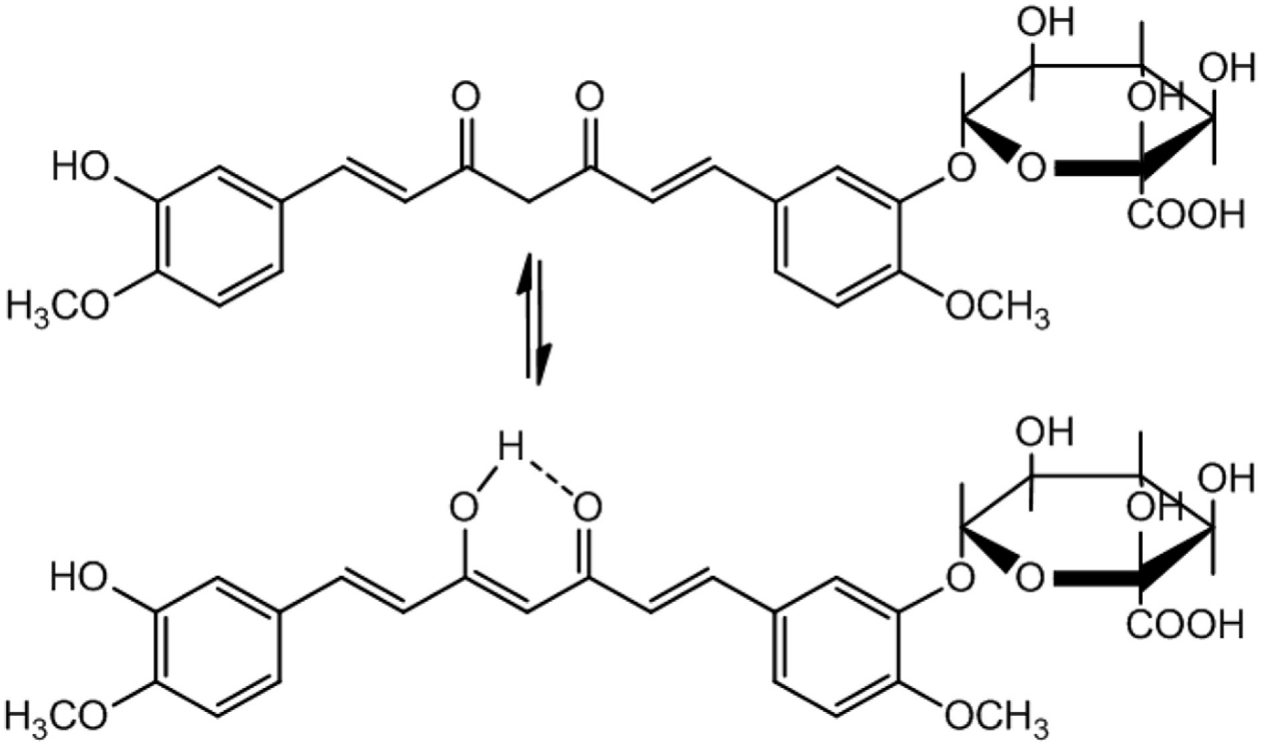
**Curcumin glucuronide: The major metabolite of Curcumin**.

A serious concern regarding pharmacological application of Curcumin is its poor pharmacokinetics (PK) profile. The bioavailability of Curcumin is low because of poor absorption, rapid elimination and/or low target organ concentration. This is due to the reason that Curcumin is conjugated when it is absorbed through the intestine, consequently free curcumin is present in extremely low level at the target organ. Moreover, in biological system it is rapidly converted to its metabolites. Nevertheless, Curcumin is non-toxic with a dose of 8g/day is well tolerable and 12g/day may cause minor adverse effects like diarrhea, headache, yellowish stools, and rashes etc. (Anand et al., 2007). Because of its low bioavailability and half-life, a small portion of the orally administered Curcumin can reach at the site of action. Therefore, Curcumin metabolites have been assumed to be responsible for its bioactivity. Curcumin glucuronide (Figure 4) was reported as the major metabolite present in the plasma after oral administration of Curcumin in rats (Shoji et al., 2014).

Recently an interesting study has been reported to compare the activity of Curcumin and its major metabolite Curcumin glucuronide (synthetic) on gene expression in HepG2 human liver cancer cell lines *in vitro*. The RT-PCR (Reverse transcription polymerase chain reaction) was used to detect the RNA expression levels. The effect of Curcumin and Curcumin glucuronide on mRNA expression of *ACOX1*, *GSTT1*, *CAT*, and *AREG* genes was studied. IL-8 expression was also evaluated by real time RT-PCR, because Curcumin inhibits IL-8 production. The results showed that the expression of all the tested genes (*ACOX1*, *GSTT1*, *CAT*, *AREG*, and *IL-8*) was significantly decreased by Curcumin treatment, whereas the effect of Curcumin glucuronide was found to be very low. Curcumin glucuronide down-regulated the expression of *GSTT1* to some extent only. In brief, the *in vitro* cell culture study suggested that Curcumin *per se* was highly active, rather than its major metabolite Curcumin glucuronide (Shoji et al., 2014).

## Colorectal (colon) cancer

Colorectal cancer is the fourth leading cause of cancer-related mortality worldwide in 2012 with a 5-years prevalent of 10.9% ^4,5^. Cortactin (cortical actin binding protein or CTTN, a monomeric protein located in the cytoplasm of cells), encoded by the *CTTN/EMS1* gene, is a v-Src substrate localized with cortical actin at the plasma membrane and is up-regulated in several types of cancer (Wu et al., 1991). The phosphorylated form of cortactin (pTyr421) plays major role in cancer cell migration and invasion. It was shown that pTyr421-cortactin was up-regulated in colon cancer. Curcumin interacted with PTPN1 tyrosine phosphatases to rise its efficacy leading to dephosphorylation of pTyr421-CTTN. Curcumin considerably reduced the pTyr421-CTTN in HCT116 cells and SW480 (adenocarcinoma of the colon) cells, but was ineffective in HT-29 (human colorectal adenocarcinoma) cells. Altogether, Curcumin modulated the activity of PTPN1 phosphatase to reduce cortactin phosphorylation and interaction with CTNND1, and finally to reduce colon cancer cell migration (Radhakrishnan et al., 2014). Curcumin activated MAPKs including Erk1/2 and JNK and inhibited phosphatases 2A (PP2A) and 5 (PP5). Up-regulation of MAPK cascade induce ROS which ultimately led to p53-independent apoptosis in cancer cells (Han et al., 2012). Curcumin inhibited cell viability in human colorectal cancer cell lines, SW480, HT-29, and HCT116 with IC_50_ values ranging between 10.26 μ M and 13.31 μ M (Cen et al., 2009). Curcumin at 10 μ M, repressed the invasive ability (22.8%) of murine colon 26-L5 carcinoma cells (Siripong et al., 2002).

## Breast cancer

The incidence of breast cancer (both genders) was second highest among all cancers (11.9%) and it rated as the leading cause of mortality due to cancer among women worldwide (fifth for both sexes) in 2012 ^4,5^. Curcumin could successfully inhibit the proliferation of human breast cancer MDA-MB-435 cells in a concentration- and time-dependent manner by accumulating the cells in the G1 phase of the cell cycle. The overexpression of *EZH2* in human breast cancer MDA-MB-435 cells caused poor prognosis in this type of cancer. The underlying molecular mechanism of Curcumin treatment was explained by the activation of the MAPK signaling pathway. Three major members of the MAPK family, the p38 kinase, JNK and ERK were stimulated to down-regulate the enhancer of zeste homolog 2 (*EZH2*) gene overexpression leading to proliferation of the cancer cells (Xu et al., 2013). Activated cancer-associated fibroblasts (CAFs) or myofibroblasts facilitate the growth of tumor as well as develop drug-resistance in human breast cancer. Hence effective therapeutic regimen should be able to inhibit the paracrine effects of these supportive cells. Treatment with Curcumin on patient-derived primary breast CAF cells overexpressed p16^INK4A^ and other tumor suppressor proteins while inactivated the JAK2/STAT3 pathway. This repressed the alpha-smooth muscle actin (α-SMA) and consequently the cell migration/invasion was reduced (Hendrayani et al., 2013).

Circulating tumor cells (CTC) are present into the vasculature from a primary tumor and flow in the bloodstream. Therefore, CTCs can be considered as seeds for subsequent metastasis in distant organs, initiating a mechanism that is responsible for the vast majority of cancer-related deaths (Gupta and Massagué, 2006). As stated earlier, invasion and metastasis are two serious problems for the prevention/treatment of cancer. The properties of circulating tumor cells (CTC) and cancer stem-like cells (CSC) are related with distant metastasis, but the mechanisms through which CSCs promote metastasis are not clear. It was reported that breast cancer cell lines with more stem-like properties display higher levels of microtentacles (McTN), a type of tubulin-based protrusion of the plasma cell membrane that forms on detached or suspended cells and aid in cell reattachment. Curcumin promptly quenched McTN in breast CSC, preventing reattachment from suspension.

Overall, a model in which breast CSCs with cytoskeletal alterations that promote McTNs can mediate attachment and metastasis but might be targeted by Curcumin as an anti-metastatic strategy (Charpentier et al., 2014). Chronic inflammation is a key risk factor for the growth and metastatic development of cancer. Curcumin inhibited the expression of the proinflammatory cytokines CXCL-1 and -2 to reduce breast and prostate cancer metastases. From the microarray miRNA expression analysis it was found that Curcumin modulated the expression of a series of miRNAs, including up-regulation of miR181b, in metastatic breast cancer MDA-MB-231 cells where miR181b down-regulated the proinflammatory cytokines CXCL-1 and -2 through a direct binding to their 3′-UTR. Curcumin-induced up-regulation of miR181b in metastatic breast cancer cells inhibited metastasis formation *in vivo* in immune-deficient mice (Kronski et al., 2014). Another study in this field suggested that metastatic development in human mammary epithelial carcinoma MCF-7 cells was inhibited by Curcumin *via* the suppression of urokinase-type plasminogen activator by NF-κB signaling pathways (Zong et al., 2012). As stated before, MMP-9 is a major factor in cancer cell invasion. An invasion-related study in MCF-7 human breast cancer cells (Kim et al., 2012a) supported that Curcumin suppressed the TPA-induced MMP-9 expression and subsequent cell invasion. The molecular mechanism involved Curcumin-induced inhibition of PKCα-dependent MMP-expression, down-regulation of NF-κB and reduction of AP-1 activation i.e., repression of the PKCα, MAPK and NF-κB/AP-1 pathway in MCF-7 cells.

It has been found that breast cancer is often associated with obesity. Most probably the relation between these two factors is mediated by adipokines. Visfatin [Nicotinamide phosphoribosyltransferase (NAmPRTase or Nampt) also known as pre-B-cell colony-enhancing factor 1 (PBEF1)] is an adipokine, that is localized to the bloodstream and has various functions. The protein visfatin has recently been shown to be related to the development and progression of breast cancer. Consequently, suppression of the gene *visfatin* might be a novel strategy to fight against breast cancer. The influence of Curcumin on *visfatin* gene suppression was investigated (Kim et al., 2012b). It was found that the mRNA and protein levels of *visfatin* were down-regulated by Curcumin in human breast cancer MDA-MB-231, MDA-MB-468, and MCF-7 cell lines. In addition, the activity of constitutive nuclear factor (NF)-κB was reduced. Taken together, *visfatin* could enhance the invasion of breast cancer cells which was down-regulated by Curcumin. Furthermore, *visfatin* knockdown by siRNA led to the reduction of cancer cell invasion. Curcumin treatment could impose anti-migratory activity in human breast cancer MDA-MB-231 cells (Chiu and Su, 2009). It inhibited proliferation and migration by increasing the Bax to Bcl-2 ratio and decreasing NF-κBp65 expression. Integrin (α6β4) is a laminin adhesion receptor with an established role in the invasion and migration of cancer cells. Curcumin successfully decreased the integrin (α6β4)-dependent breast cancer cell motility and invasion in a dose-dependent manner without affecting apoptosis in MDA-MB-435/β4 (β_4_-integrin transfectants) and MDA-MB-231 breast cancer cell lines (Kim et al., 2008).

*Maspin* (mammary serine protease inhibitor) can suppress tumor growth and metastasis *in vivo* and tumor cell motility and invasion *in vitro* in breast cancer. The maspin expression in Curcumin-treated MCF-7 (wild type p53) at transcription and translation levels was analyzed by RT-PCR, immunofluorescence, and Western blotting. The results showed a correlation of maspin expression with p53 and Bcl-2 levels. Curcumin inhibited cell proliferation by inducing apoptosis and up-regulation of *maspin* gene expression was observed in MCF-7 cells. These findings were further correlated with the up-regulation of p53 protein and down-regulation of Bcl-2, suggesting maspin mediated apoptosis in MCF-7 cancer cells (Prasad et al., 2010). These findings were further correlated with the up-regulation of p53 protein and down-regulation of Bcl-2, suggesting maspin mediated apoptosis in MCF-7 cancer cells (Prasad et al., 2010). The chemopreventive activity of Curcumin in transformed breast cells was investigated (Kim et al., 2001). Curcumin inhibited H-ras-induced invasive phenotype in MCF10A human breast epithelial cells (H-ras MCF10A); repressed MMP-2 and exerted cytotoxic effect on H-ras MCF10A cells in concentration-dependent manner. The apoptotic cell death involved significant down-regulation of Bcl-2 and up-regulation of Bax. Curcumin treatment resulted in the generation of ROS in H-ras MCF10A cells. In brief, the results supported that curcumin inhibited invasion and induced apoptosis in the transformed breast cancer H-ras MCF10A cells. Plectin can play a pivotal role in the migration and invasion of human MDA-MB-231 breast cancer cells. The down regulation of plectin gene *PLEC1* by siRNA promotes the migration and invasion of the MDA-MB-231 cancer cells. The plectin siRNA has been reported (Ha et al., 2011) to alleviate by curcumin.

Epithelial-mesenchymal transition (EMT), a fundamental procedure of embryogenesis, is a process by which epithelial cells gain migratory and invasive abilities by down regulation of the proteins such as E-cadherin and γ-catenin (subsequent loss of cell polarity and cell to cell adhesion) and cells may acquire mesenchymal markers such as MMP-2, and -9, N-cadherin, fibronectin, vimentin to obtain enhanced ability for cell migration and invasion. After migrating to a suitable site the tumor cells up regulate E-cadherin and other epithelial markers through a process known as MET (mesenchymal-epithelial transition). Curcumin showed its ability to inhibit lipopolysaccharide (LPS) prompted EMT and corresponding morphological changes in MCF-7 and MDA-MB-231 human breast cancer cell lines. It down-regulated the LPS-induced markers of EMT such as vimentin through the downregulation of NF-κB-Snail activity and up regulated the expression of E-cadherin. The obvious outcome was inhibition of cell motility and invasiveness in human breast cancer cell lines *in vitro* (Huang et al., 2013). A study related to the effect of curcumin in ER-negative human breast cancer MDA-MB-231 cells revealed that the anti-invasive effect of curcumin was independent of the presence of estrogen. Curcumin regulated two common biomolecules MMP-2 (downregulation) and TIMP-1 (upregulation). It also successfully reduced the transcript levels of two major angiogenesis factors VEGF and b-FGF (Shao et al., 2002).

## Prostate cancer

The incidence of prostate cancer was 7.9% of all cancers worldwide in 2012 with a mortality rate of 3.7% and comparatively higher (12.1%) five-years prevalent ^4,5^. Curcumin inhibited the androgen independent human prostate cancer DU145 and PC3 cell proliferation in a dose-dependent manner and showed its activity to decrease extensively the cell migration as well as the detachment of cells seeded on laminin or fibronectin by selectively activating the ERβ, the only isoform present in these prostate cancers. Hence curcumin might be an effective agent against human prostate cancer (PCa) progression. Therefore, the estrogenic effect of curcumin might be protective against PCa invasion and metastasis (Piccolella et al., 2014). VEGF expression and secretion are well-correlated with the levels of Osteopontin (OPN) in PC3 (a human bone-derived androgen-independent prostate cancer) cells. Phosphorylation of ERK1/2 associated osteopontin/αvβ3 signaling pathway regulates the expression of VEGF. Curcumin significantly repressed the phosphorylation of ERK1/2 and expression of VEGF. It also inhibited the activation of MMP-9 which could down regulate the secretion of VEGF165b (an anti-angiogenic factor) and angiostatin (a potent inhibitor of angiogenesis and an important factor to suppress the growth of secondary tumors in mice bearing previous tumors) (Gupta et al., 2013a).

CCL2 (CC motif ligand 2, also known as monocyte chemo attractant protein-1 or MCP-1) is a small cytokine that belongs to the Chemokines-chemotactic cytokines family, plays a pivotal role in the development and progression of PCa bone metastasis (Lu et al., 2006). Curcumin at 30 μ M inhibits adhesion, invasion, and motility of the human PC-3 cells, to some extent through the down-regulation of CCL2 activity via the inhibition of PKC and MMP-9 (Herman et al., 2009). It also broadly down regulated the mRNA expression in PC3 cell lines *in vitro*.

## Brain and nervous system cancer

Brain and nervous system cancer counted 2.3% cancer related deaths worldwide in 2012 with a poor five-years prevalent (0.9%)^4,5^. A major problem for the treatment of brain cancer is the presence of blood brain barrier (BBB). Because of this reason many frontline antiproliferative, anti-invasive, anti-metastatic, and anti-angiogenic drugs cannot work at all in brain cancer. Curcumin reduced the proliferation of mouse-rat hybrid retina ganglion N18 cells through cell cycle (G2/M phase) arrest and induction of apoptosis. Moreover, it inhibited the invasion and migration of N18 cells in time- and dose-dependent manner *in vitro*. The Western blot study revealed that curcumin efficiently down regulated the protein levels of PKC, FAK, NF-κB p65 and Rho A leading to the inhibition of ERK1/2, MKK7, COX-2, and ROCK1 respectively. The inhibition of cell proliferation might be because of repression of NF-κB p65 and consequently COX-2 whereas the down regulation of PKC, FAK, and Rho A as well as their downstream targets ERK1/2, MKK7, and ROCK1 led to the overall down regulation of MMP-2 and -9, responsible for N18 cancer cells migration and invasion (Lin et al., 2010). Curcumin demonstrated anti-proliferative, anti-migratory, and anti-invasive characteristics against in five human glioblastoma (GBM) cell lines *in vitro*. Two human primary glioblastoma cell lines A-172 and MZ-18 as well as three recurrent GBM (MZ-54, MZ-256, MZ-304) cell lines were considered for study (Senft et al., 2010). To evaluate cell proliferation, migration and invasion of the five GBM cell lines; cell growth assays, monolayer wound healing assays and modified Boyden chamber trans-well assays were conducted at different concentrations. Curcumin reduced the cell growth of the GBM cells in time- and concentration-dependent manner. The sandwich-ELISA test specified the levels of the transcription factor phospho-STAT3, a potential target of curcumin. The effect of curcumin was mediated, at least in part, by interference with the STAT3 signaling pathway.

## Nasopharyngeal cancer

Nasopharyngeal cancer caused 0.6% mortality due to cancer worldwide in 2012 with a poor five-years prevalent (0.7%) ^4,5^. In pharyngeal carcinoma, mucositis is developed during the administration of anticancer drugs or radio therapy. It causes delays in subsequent chemotherapy cycles which may require alteration of dose (reduction) and/or therapeutics. This alteration very often carries negative influence on overall survival of the patients. Curcumin is a strong inhibitor of NF-κB activation and subsequent cytokine release. Preincubation of human pharyngeal carcinoma Detroit 562 cells with 200 μ M Curcumin for 5–60 min resulted in complete suppression of the release of tumor necrosis factor-α (TNF-α), interleukin (IL)-6, IL-8, monocyte chemoattractant protein 1 (MCP-1), granulocyte macrophage-colony stimulating factor, and VEGF. Repetitive exposure to curcumin resulted in repetitive suppression of cytokine/chemokine expression lasting from 4 to 6 h. Most interestingly, the two wound-healing cytokines (FGF-2 and interferon-γ) were not inhibited by curcumin which clearly indicates the selectivity of curcumin in this process (Luer et al., 2011, 2012). Thus, curcumin showed strong antibacterial effect against a facultative upper respiratory tract pathogen by inhibiting bacterial growth, adherence, invasion, and proinflammatory activation of upper respiratory tract epithelial cells *in vitro*.

## Bone cancer

Besides the above, curcumin displayed its anti-invasive and anti-metastatic potential against human osteosarcoma *in vitro*. Osteosarcoma is an aggressive primary malignancy of the bone, predominantly affects rapidly growing bones in adolescents and children, is characterized by locally aggressive growth and early metastatic potential arising from primitive transformed cells of mesenchymal origin (and thus a sarcoma)—that exhibits osteoblastic differentiation and produces malignant osteoid generating defective immature bone or osteoid (Luetke et al., 2014). It predominantly affects children and adolescents and shows an aggressive tendency for growth and metastasis, with as many as 20% of patients reported to have developed pulmonary metastasis at the time of diagnosis. Regardless of extensive uses of chemotherapy, radiotherapy and surgery, respiratory failures due to lung metastases are common events and the major cause of death in these patients. Curcumin inhibited the cell proliferation in human osteosarcoma U2OS, SaOS-2 and HOS cells in a concentration-dependent manner and its anti-invasive potential in human osteosarcoma U2OS cells was studied. Curcumin displayed its ability to regulate the Wnt/β-catenin signaling pathway. It considerably reversed β-catenin plasmid mediated increase in invasive capacity of U2OS cells in a dose-dependent manner (Leow et al., 2010, 2014). Other than osteosarcoma, Curcumin showed its anti-invasive and anti-metastatic capabilities in fibrosarcoma (or fibroblastic sarcoma), a different type of bone cancer, usually found in males at the age of below 40 tears. In fibrosarcoma, the malignant mesenchymal tumor originates from fibrous connective tissue (also periosteum and overlying muscle) and recognized by the presence of immature proliferating fibroblasts or undifferentiated anaplastic spindle cells in a storiform pattern. If all grades are included, primary fibrosarcoma of the bone has a worse prognosis than osteosarcoma. It is well known that the net balance of MMPs and tissue inhibitor of metalloproteinases (TIMPs) system plays a key role in tumor cell invasion. The effect of curcumin on the molecular mechanisms of anti-invasive and antimigratory activity of transforming growth factor (TGF)-β1 in HT1080 human fibrosarcoma cells was investigated. Curcumin successfully down regulated the MMP-2, -9 and TIMP-1, -2 to maintain the net balance of MMPs/TIMPs to inhibit, at least in part, cancer cell invasion and migration (Kwak et al., 2006) in HT1080 human fibrosarcoma cancer cells.

## Conclusion

Curcumin is a cheap, non-toxic, and easily available natural polyphenol with excellent medicinal and commercial demands. Many costly products can be derived very easily from curcumin. For example, Curcumin can easily be converted to vanillin through a single-step conversion (Bandyopadhyay and Banik, 2012). A huge number of preclinical and early-phase clinical studies undoubtedly confirmed this nutraceutical as safe with vast potential of becoming an effective anti-invasive and anti-metastatic chemotherapeutic, possibly in combination with other drugs and/or appropriate structural modification for the treatment of cancer. The only concern about this compound is its poor ADME profile. The bioavailability of curcumin is low because of poor absorption, rapid elimination and/or low target organ concentration. On the other hand, curcumin exerted multiple anticancer properties *in vitro*, *ex vivo* and *in vivo* as well as in clinical trials by regulating a variety of biological pathways involved in tumor invasion, metastasis and angiogenesis. Based on its huge therapeutic aspects several active investigations are going on to overcome the ADME-related drawbacks by introducing new and novel formulation and route of administration to achieve the highest therapeutic level. It is highly expected that Curcumin, the golden spice of India, with novel formulation/route of administration will *metastasize* from *curry to capsule* for the treatment of cancer invasion and metastasis.

### Conflict of interest statement

The author declares that the research was conducted in the absence of any commercial or financial relationships that could be construed as a potential conflict of interest.

## Footnotes

^2^http://www.cancer.org/cancer/cancerbasics/thehistoryofcancer/the-history-of-cancer-what-is-cancer (Accessed on August 30, 2014).

^3^http://www.cancer.gov/researchandfunding/priorities/global-research-activities (Accessed on September 04, 2014).

^4^http://www.who.int/mediacentre/factsheets/fs297/en (Accessed on September 04, 2014).

^5^http://globocan.iarc.fr/Pages/fact_sheets_cancer.aspx (Accessed on September 04, 2014).

^6^http://www.cancer.gov/cancertopics/understandingcancer/cancer/UNDERCAN.PDF (Accessed on August 30, 2014).

^7^http://www.accessdata.fda.gov/scripts/fcn/fcnNavigation.cfm?filter=turmeric&sortColumn=&rpt=grasListing (Access date: September 04, 2014).

^8^Spices Board of India. Ministry of Commerce and Industry. Available online: http://www.indianspices.com (Access date: September 04, 2014).

## Abbreviations

ADME: Absorption, distribution, metabolism, excretion
APN: Aminopeptidase N
BBB: Blood brain barrier
b-FGF: basic fibroblast growth factor
Cav-1: Caveolin-1
CCL2: Chemokines cytokine motif ligand 2
CD: Cluster of differentiation
COX-2: Cyclooxygenase-2
EGFR: Epidermal growth factor receptor
EMT: Epithelial-mesenchymal transition
ER: Estrogen receptor
ERK1/2: Extracellular signal-regulated kinases 1 and 2
FAK: Focal adhesion kinase
FGF-2: Fibroblast growth factor-2
GBM: Glioblastoma
GM-CSF: Granulocyte macrophage-colony stimulating factor
HCC: Human hepatocellular carcinoma
IC_50_: Half-maximal inhibitory concentration
LPS: Lipopolysac charide
MCP-1: Monocyte chemoattractant protein 1
MMP: Matrix metalloproteinase
mRNA: messenger ribonucleic acid
NF-κB-Snail: Nuclear factor κB-Snail
NSCLC: Non-small cell lung carcinoma
OPN: Osteopontin
PCa: Prostate cancer
PKC: Protein kinase C
Rho A: Ras homolog gene family, member A
ROCK1: Rho-associated, coiled-coil-containing protein kinase 1
ROS: Reactive oxygen species
RT-PCR: Reverse transcription polymerase chain reaction
SCLC: Small cell lung carcinoma
siRNA: small interference RNA
STAT: Signal transducer and activator of transcription
TGF-β1: Transforming growth factor-β
TIMP-1: Tissue inhibitor of metalloproteinase-1
TNF-α: Tumor necrosis factor-α
VEGF: Vascular endothelial growth factor
VM: Vasculogenic mimicry.
